# Microarchitecture of historic bone samples with tuberculosis

**DOI:** 10.1007/s00508-022-02017-y

**Published:** 2022-03-20

**Authors:** György Vekszler, Matthias Granner, Elena Nebot Valenzuela, Eduard Winter, Martin Dockner, Gerhard W. Weber, Michael Pretterklieber, Maria Teschler-Nicola, Peter Pietschmann

**Affiliations:** 1 Institute of Pathophysiology and Allergy Research, Center for Pathophysiology, Infectiology and Immunology. Medical University of Vienna, Vienna, Austria; 2 Karl Landsteiner University of Health Science, Krems, Austria; 3 Department of Physiology, Faculty of Medicine, Complutense University of Madrid, Madrid, Spain; 4 Pathological-Anatomical Collection in the Fool’s Tower, Department of Anthropology, Natural History Museum Vienna, Vienna, Austria; 5 Department of Evolutionary Anthropology, University of Vienna, Vienna, Austria; 6 Core Facility for Micro-Computed Tomography, University of Vienna, Vienna, Austria; 7 Gottfried Schatz Research Center, Division of Anatomy, Medical University of Graz, Graz, Austria

**Keywords:** Mycobacterium tuberculosis, Bone structure, Cortical defects, Ankylosis, Historical pathological anatomical collection

## Abstract

Tuberculosis is among the leading causes of death from infectious diseases and affects many organ systems, including the skeleton. Skeletal tuberculosis is an extrapulmonary stage of tuberculosis, which occurs after the early and post-primary pulmonary stages of the disease. The aim of our study was to assess the microarchitecture of historic dry bone samples of subjects who have died of tuberculosis documented by post-mortem examinations. These preparations date to the pre-antibiotic era, and were provided by the Pathological-Anatomical Collection in the “Fools Tower” of the Natural History Museum Vienna (PASiN-NHM).

We investigated macerated samples of 20 vertebral bodies, 19 femoral heads, and 20 tibiae of a total of 59 individuals diagnosed with tuberculosis from the nineteenth and early twentieth century. 10 femora and 10 tibiae from body donors that did not exhibit signs of infection and 10 (unaffected) vertebrae kept at the PASiN-NHM were studied as controls. The affected regions of the bone samples (and the corresponding regions of the control bones) were analyzed by microcomputed tomography using a Viscom X 8060 II system. Obtained images were analyzed semi-quantitatively. In samples with tuberculosis, independent of the investigated skeletal region, trabecular defects and decreased trabecular thickness were observed. Cortical porosity was seen in affected vertebrae and tibia; in tuberculous tibiae (but not in the femora) cortical thickness was decreased. In half of the individuals, cortical sclerosis was present; signs of ankylosis were observed mainly at the femoral heads affected with tuberculosis. We conclude that a combination of several alterations at the trabecular compartment could be suggestive of the presence of tuberculosis in historic skeletal remains.

**Supplementary Information:**

The online version of this article (10.1007/s00508-022-02017-y) contains supplementary material, which is available to authorized users.

## Introduction

Tuberculosis is an ancient infectious disease; it had been suggested that a tuberculosis-like disease was already present in a 245-million-year-old *Proneusticosaurus silesiacus* [1]. The oldest case of tuberculosis infection in humans has been identified based on multiple approaches in a Levantine Early Neolithic population approximately 9000 years before present (BP) [2]. Further cases of infection caused by *Mycobacterium tuberculosis* were also reported from periods such as the Copper, Bronze and Iron Ages [3, 4]. In the Middle Ages, the infection rate increased significantly because of the growing population density and poor living conditions [5, 6]. In the nineteenth century tuberculosis was a widespread disease, which was almost eradicated in Europe in the twentieth century. This was due to the improving hygienic and social conditions as well as successful treatment and prevention. In the 21st century, we are seeing a resurgence in the number of patients with tuberculosis According to data published by the World Health Organization (WHO), there are currently 20 million people with active tuberculosis worldwide. Annually, 8 million new cases are registered [6–10].

Approximately 1.5 million people die from this disease each year. Currently it is ranked number nine of the most common causes of death, with the second highest death number of all infectious diseases, ahead of acquired immunodeficiency syndrome but ranking behind coronavirus disease 2019 [11].

Skeletal tuberculosis is an extrapulmonary stage of tuberculosis, which occurs after the early and post-primary pulmonary stages of tuberculosis. Skeletal tuberculosis only accounts for approximately 3% of all cases, while extrapulmonary tuberculosis represents about 30% of the total number of cases [12]. The most common site for skeletal involvement is the spine (Pott’s disease), accounting for almost 50% of all the cases, followed by the pelvis and hip in 20%, and femur (10%), knee and tibia (10%), ribs (7%), and multiple other sites (3%). [12, 13]. Today, the timely administration of antibiotics usually prevents the progressive development of skeletal tuberculosis; moreover, immunization with Bacillus Calmette-Guérin (BCG) vaccination can additionally help to prevent the disease [14, 15].

Within the last few years, the use of microcomputed tomography (micro-CT) to assess trabecular and cortical bone morphology for clinical diagnostic reasons has grown immensely [16, 17]. In contrast to bone histology, it is a non-invasive approach with many advantages: no tissue destruction, and, to a certain extent, a good substitute for the more laborious histological investigation. Furthermore, while histological examination provides only two-dimensional images, computed tomography enables 3D-visualization of structures and better assessment of spatial relationships. In addition to computed tomography, microcomputed tomography has also been successfully used for several years in paleopathology and evolutionary biology [18–21]. Among others, our research group previously used this approach for the analysis of historical samples affected by bone diseases such as Paget’s disease and osteomyelitis [22, 23].

The present study aimed to examine microstructural bony alterations of historical samples taken from individuals who suffered from late-stage tuberculosis according to the patient’s medical history. The specimens, dry preparations, housed at the Pathological-Anatomical Collection in the “Fools Tower” of the Natural History Museum Vienna (PASiN-NHM) were taken in the course of postmortem examinations in the nineteenth and early twentieth centuries, and, thus, stem from the pre-antibiotic era. Our goal was to evaluate and analyze semi-quantitatively the microarchitecture of trabecular and cortical bone alterations of affected vertebral bodies (representing Pott’s disease), ankylotic hip joints or single femoral heads (representing hip joint tuberculosis), and tibiae (representing an element of knee joint tuberculosis), and to compare them with unaffected, healthy specimens.

## Material and methods

### Samples

We examined 20 vertebral bodies of 20 individuals (1 cervical, 3 thoracic, 11 lumbar, and 2 sacral, 3 not identified). Nineteen femoral heads (including 13 preparations of the hip) of 19 individuals and 20 tibiae (including 12 knee preparations) of 20 individuals stored at the Pathological-Anatomical Collection in the “Fools Tower” of the Natural History Museum Vienna (PASiN-NHM). With regard to the tibiae, 18 proximal and two distal parts were affected and therefore assessed. The specimens were dry preparations and were categorized under the clinical-pathological diagnosis “tuberculosis” based on a synopsis between the pathological findings and the clinical records.

The Supplemental Tables (Supp. Tab. 1–3) provide an overview of the biographical data (sex, age at death, year of death) and the body regions included in the study. Supplemental Table 1 shows that out of 20 analyzed vertebral specimens, 3 were from females and 2 from males. There was no information about the 15 other samples. The deceased with a known record were born between 1797 and 1917; the median age of the deceased was 25 years. The age at death ranged from 10 to 37 years.

As detailed in Supplemental Table 2, of a total of 19 affected femora, 7 cases (37%) were from the left and 12 cases (63%) were from the right side. Ten (53%) specimens were obtained from males, there was no information concerning the sex of the individual in the nine (47%) remaining specimens included. The age at death ranged from 12 to 66 years. The median of age of the cases with a known record was 24 years. The deceased were born between 1798 and 1871.

As shown in Supplemental Table, 3, out of 20 analyzed tibial specimens, in 5 (20%) the right side, and in 9 (45%) the left side was affected. In six (30%) specimens we could not unequivocally identify the laterality due to deformity and defects. Three specimens were from females, one from a male; there was no information about the 16 other specimens. The median age of the deceased with an available record was 53 years. They were born between 1834 and 1902. The age at death ranged from 25 to 67 years.

### Control bones

The microstructure of the femora and tibiae with tuberculosis was compared to that of 10 femora and 10 tibiae from recent body donors provided by the Division of Anatomy, Medical University of Vienna used in a previous study [22]. The mounted vertebral columns presenting signs of tuberculosis also contained macroscopically unaffected vertebrae. Ten of these vertebrae were used as controls.

### Microcomputed tomography

All bones samples were scanned with a Viscom X 8060 NDT (Viscom AG, Hannover, Germany), a generic X‑ray inspection system for non-destructive qualitative visualization at the Vienna Micro-CT Laboratory. The specimens were scanned using the following parameters: transmission tube, digital detector, 120 kV, 310 µA, filter 0.50 mm copper. In order to achieve a spatial resolution of 50 µm, a zoom factor in the range of × 3.5 was used.

The regions of interest (of the diseased bones) were defined as follows: the complete vertebral bodies, the proximal part of the femur including the trochanter minor, the proximal part of the tibia including the tibial tuberosity, the distal part of the tibia including the syndesmosis tibiofibularis. The cortical and trabecular structures of the obtained images were analyzed semi-quantitatively by comparing the diseased bones and the corresponding regions of the control bones.

Trabecular thickness, trabecular number, trabecular separation, trabecular and cortical defects, cortical thickness, cortical porosity, cortical trabecularization, spongiform transformation of cortical structures, sclerosis and signs of ankylosis were assessed. If applicable, we followed the recommendations of the Nomenclature Committee of the American Society of Bone and Mineral Research for our terminology [24]. Thus, “cortical porosity” was defined as an increased radiolucency of cortical structures. The term “trabecularization” describes an increase of pores and inhomogeneity of the cortices; “spongiform transformation” is defined as the complete replacement of cortical by trabecular structures. “Sclerosis” describes a decreased radiolucency of structures; the term “ankylosis” indicates the complete loss of an articular space (involving also the cortical articular structures). Every parameter of each specimen was assigned to one of the seven values that may vary between “−−−” and “+++” according to the severity relative to the control bones (the ranges were: −−−, −−, −, 0, +, ++, +++).

The semi quantitative analysis was carried out twice by the same investigator (GV), considering a 2-week interval. After the second assessment, their values where compared. If the two assessments came to the same result, the assigned value was used. If the results diverged, another investigator (PP) assessed the specimen and an agreement on the value to be used was achieved. The data obtained are described as individual values and medians.

## Results

### Macroscopic features

All samples displayed typical macroscopic features of bone tuberculosis [24–26]. When comparing the samples to healthy bones, a brittle bone quality, pursuant to osteoporosis, became visible. Typically, bone defects of different sizes were seen; in many cases the cortical bone was extremely thinned. In one mounted vertebral column and one tibia, sequester formation was observed.

In some vertebral bodies, defect sites without cover plate collapses or axis misalignment were visible. In contrast, in other specimens the vertebral bodies were destroyed in a way that the individual segments could no longer be identified macroscopically. Most spinal column specimens showed axial misalignments presumably due to defects and ankyloses. Pott’s deformity was present in 6 out of the 20 spine preparations.

Macroscopically, several of the vertebral segments and the majority of hip specimens showed ankylosis; the ankylotic joints presented a uniform bone structure lacking any segment boundary. According to the signs of ankylosis present at the femoral heads, the affected hip joints must have been fixed in different positions. Thus, the mobility of the joint must have been completely eliminated.

### Bone microarchitecture

A representation of the semi-quantitative assessment of trabecular and cortical bone structure is shown in supplemental tables 4–9.

The following table (Table 1) shows the median of semi-quantitative assessment of trabecular and of cortical microarchitecture in vertebral body, femoral head and tibia samples with tuberculosis.

**Table 1:** Median of semi-quantitative assessment of trabecular (Tb.) and of cortical (Ct.) microarchitecture in vertebral body, femur and tibia samples with tuberculosis. Symbols indicate the direction (+: increase, 0: no change, −: decrease) and the severity (e.g. +, ++, +++) of alterations

	Vertebral body samples	Femur samples	Tibia samples
	Median	Median	Median
*Tb. thickness*	− −	–	− − −
*Tb. number*	+	0	+ + +
*Sclerosis*	0/+	+	0/+
*Tb separation*	0	+	0
*Tb. defect*	+ +	+	+ +
*Ankylosis*	0/+	+ +	0
*Ct. thickness*	− −	+	− − −
*Ct. porosity*	+ +	+	++/+++
*Trabecularization*	0	+	+ +
*Ct. defect*	+ +	+	+
*Sclerosis*	0	+	0
*Spongy transformation*	0	+	+ +

**Fig. 1:**
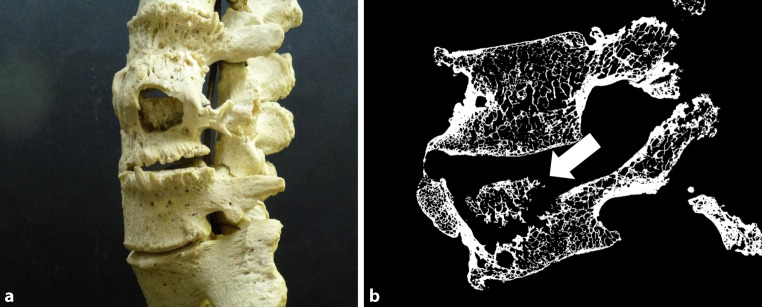
Tuberculous spine segment including the second to fifth lumbar vertebrae (L2–L5) in left lateral view; there was no information about age and sex and year of death. The corresponding microcomputed tomography (micro-CT) image is oriented in the sagittal plane. Both macroscopically (**a**) and on the micro-CT (**b**), one can see a decrease in height of the L2 vertebral body, more pronounced in the area of the vertebral canal. The structures of L3 are immensely destroyed. Osteophyte formation is visible on all segments. The sequester inside the L3 vertebral body (*arrow*) is surrounded by a pronounced defect zone and a sclerosis zone. This picture shows a typical stage of bone tuberculosis representing the impeding collapse of the vertebral body leading to a Pott’s gibbus. Inventory number of the collection: MN 25.730

**Fig. 2:**
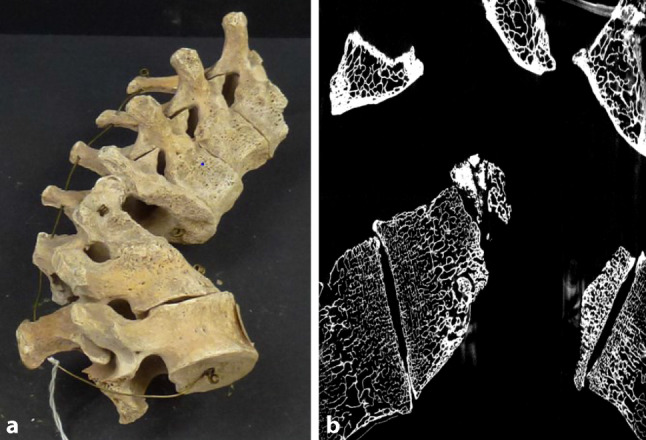
**a** Pott’s disease of the thoracolumbar segment of a patient who died in 1924. The macroscopical view shows a gibbus and progressed destruction of the vertebral bodies. **b** The micro-CT image shows a sagittal view of the thoracolumbar segment with a complete destruction of two vertebral bodies and a reduced trabecular and cortical thickness of the vertebral bodies. The cover plate of the second vertebra has been completely removed. The remaining part of the vertebral body shows a sharp defect border within the cancellous substance. Inventory number of the collection: MN.17.747/634 B20

**Fig. 3:**
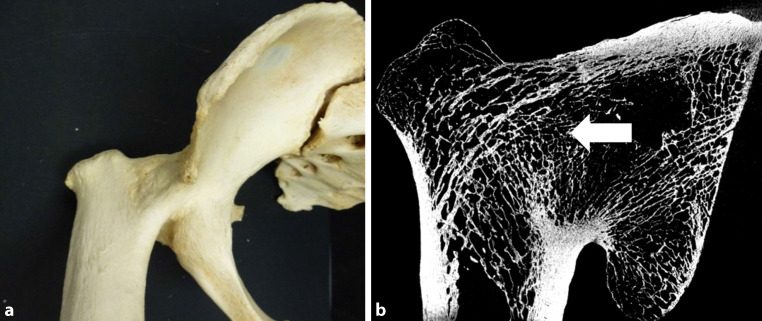
**a** Tuberculous arthritis of the ankylotic right hip joint of a 66 year old male who died 1937. The joint is fixed in a 90° flexion position and apparently completely ossified. Neither the femoral head nor the acetabulum can be identified. We can see a pronounced bone defect in the middle of the ankylotic joint. **b** The micro-CT image (in horizontal view) of the ankylotic hip shows remodeling of the trabecula (*arrow*). Inventory number of the collection: MN1639

**Fig. 4:**
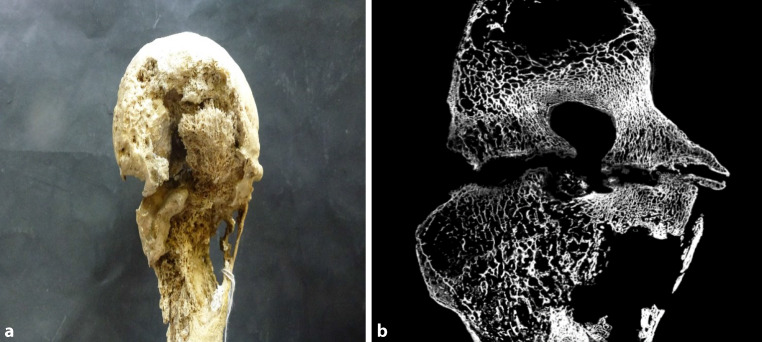
**a** Tuberculous left knee joint (anterior view); there was no information about age of death, sex or year of death. Macroscopically, one sees a destruction of both femoral condyles with sequester formation. By incomplete ankylosis, pronounced on the medial aspect, the joint is fixed in 90° flexion position. Development of osteophytes and destruction of the bone structure of the tibia can be observed in the micro-CT (**b**). The medial condyle of the tibia is almost completely dissolved

Trabecular thickness was reduced in all skeletal parts – femora, tibiae, vertebrae – of individuals that suffered from tuberculosis compared to the control group; the changes were most pronounced at the (proximal) tibia and least pronounced at the femoral head. Trabecular number was clearly increased at the tibia and slightly elevated at the vertebrae; in contrast, the median trabecular number of the femora was comparable to the control group. Trabecular separation was slightly increased at the femur. The majority of samples showed sclerotic changes at the trabecular compartment. Trabecular defects were seen in almost all specimens affected by tuberculosis (Figs. 1, 2, 3 and 4), but in none of the control bones.

Cortical thickness was reduced in the tibiae and the vertebral bodies, whereas in the femurs the median cortical thickness was slightly increased. Cortical porosity was present in almost all affected bone preparations, mostly in the tibia, followed by the vertebral body, and least at the femoral head. Trabecularization of cortical structures was mostly seen in the tibia, followed by the femoral head. There was no spongy transformation in the vertebral bodies; spongiform transformation of cortical structures occurred more often in the tibia than at the femur. Cortical defects were observed in most specimens affected by tuberculosis (Figs. 1, 2, 3 and 4); in approximately 50% of the preparations cortical sclerosis was present. In half of the specimens ankyloses were seen, which was particularly frequent at the femur (Fig. 3). Inner corticalization was seen in three tibia samples but could neither be observed in the femoral head nor the vertebral body.

## Discussion

This study for the first time investigated a series of vertebrae, femora and tibiae as well as entire hip and knee joints preserved in a historic collection that are affected by tuberculosis using microcomputed tomography (micro-CT). In the trabecular compartment of all three investigated sites, trabecular defects and decreased trabecular thickness were observed. Cortical porosis and cortical defects were seen at all sites, whereas more femora than tibiae showed ankylosis.

Already in 2007 Rühli and colleagues [19–21] have convincingly shown the diagnostic potential of micro-CT image technique compared to histological techniques to asses pathological alterations of historical human (skull) skeletal remains. Hitherto there were several studies presented that applied this non-invasive technique for the qualitative assessment of pathological skeletal alterations [16–20], including two reports on tuberculosis: Kuhn et al. compared micro-CT to histology in a pathology reference series that also contained one specimen of the hip joint severely affected by tuberculosis [18], In this case, both methods yielded complementary results implying that micro-CT can successfully be applied for the identification of assumed cortical lesions. Niklisch et al. analyzed bones excavated in Saxony-Anhalt (mainly neck of the ribs) from the early Neolithic period (5400–4800 BC) [27]. Niklisch’s samples were not only macroscopically examined by micro-CT, but by histology and molecular biology as well. Hereby, bony appositions consisting of several layers of new bone formation were detectable on the inner aspect of 14 ribs out of 118 samples. These alterations were likely caused by pleurisy, an unspecific sign of tuberculosis [5, 6, 28–30].

In contrast to the aforementioned osteoblastic rib lesions, the alterations observed in our study were caused by haematogenous spread of the disease. In accordance with the literature on macroscopic characteristics of tuberculosis [24], we observed lytic lesions (i.e. cortical and trabecular defects) of the affected bones. In addition to these defects, our findings of decreased trabecular thickness and trabecularisation of cortical structures point towards a preponderance of bone resorption over bone formation.

There are several cellular and molecular studies that may explain the mechanism of these alteration of bone metabolism. Already in 1997, the mycobacterium tuberculosis heat shock protein chaperonin 10 was reported to increase bone resorption and to prevent the proliferation of the osteoblasts. Hotokezaka et al. infected an osteoblast-like cell line (MC3T3-E1) with *Mycobacterium bovis* Bacillus Calmette-Guerin and observed a dose-dependent reduction of the proliferation and alkaline phosphatase activity of the MC3T3-E1 cells [31]. Interestingly this study also reported that infected MC3T3-E1 cells exhibited an increased production of interleukin (IL)-6, a cytokine that increases bone resorption. Liu and co-workers reported an increased number of osteoclasts in human osteoarticular tuberculosis samples and suggest that tumor necrosis factor‑α inhibits the apoptosis of *Mycobacterium tuberculosis* infected osteoclasts by regulating their autophagy [32]. Another cytokine that plays a role in *Mycobacterium tuberculosis* infection is IL-32. [33]. IL-32 increases bone resorption but also appears to be responsible for excessive bone formation seen in ankylosing spondylitis [34]. It is therefore tempting to speculate that an excessive expression of IL-32 could also be responsible for the ankylotic alterations seen in our study. The fact that in many samples not only osteolytic but also ankylotic alterations were seen indicates that patients have survived bone tuberculosis for at least several months. This is illustrated by Fig. 3 showing a complete remodelling of the ankylotic region and a dissolution of the joint line. The development of such alterations appears to be related to the infectious processes that first destroy joint structures and then, through the process of bone formation, result in ankyloses. In contrast, according to trauma surgery experience, femoral neck fractures may lead to the formation of pseudoarthroses due to the interruption of blood supply and the following aseptic femoral head necrosis (so-called Girdlestone’s hip).

In 1779 the English surgeon Percival Pott showed that in tuberculosis the intervertebral joints are not dislocated, but rather that a destructive process occurs within the vertebral bodies. Pott’s name in later centuries is associated with tuberculous vertebral destruction in the terms Pott’s disease and Pott’s hump. The presence of Pott’s disease now is regarded as a classical feature of tuberculosis [35–38]. In contrast to tuberculosis, collapse of a vertebral body in actinomycosis is rarely seen [39, 40]. Our findings of trabecular defects and trabecular thinning clearly establish a basis for vertebral fragility in tuberculosis. Schamall et al. investigated two cases of actinomycosis by macroscopy, histology and micro-CT and found new periosteal bone deposition and the presence of plexiform bone [41].

Our findings on the microstructure of skeletal tuberculosis could be helpful for the differential diagnosis of bone alterations observed in historic skeletal remains. Until now, our expertise in the application of microcomputed tomography in paleopathology includes osteomyelitis, syphilis, actinomycosis and Paget’s disease (Table 2 and 3). In historical bone samples diagnosed with (unspecific) osteomyelitis Lamm et al. observed cortical thinning and cortical porosity but, in contrast to our samples with tuberculosis, did not observe alterations of the trabecular compartment [23].

**Table 2:** Overview of bony alterations of trabecular structures in tuberculosis, syphilis, actinomycosis osteomyelitis and Paget’s disease in comparison to healthy bone (–: no information available)

Disease	Region	Thickness	Number	Sclerosis	Trabecular separation	Trabecular defect	Ankylosis	Reference
*Tuberculosis*	Vertebrae	Decrease	Slight increase	No difference	No difference	Increased	Slight increase	This study
*Tuberculosis*	Femur	Slight decrease	No difference	Slight increase	Slight increase	Slight increase	Increased	This study
*Tuberculosis*	Tibia	Decreased	Increased	Slight increase	No difference	Increased	No difference	This study
*Syphilis*	Skull	**-**	**-**	**-**	**-**	**-**	**-**	[42]
*Actinomycosis*	Lumbar vertebrae	**-**	**-**	**-**	**-**	Increased	**-**	[41]
*Actinomycosis*	Pelvic ring	**-**	**-**	**-**	**-**	Increased	**-**	[41]
*Osteomyelitis*	Femur	**-**	**-**	**-**	**-**	**-**	**-**	[23]
*Paget’s disease*	Femur	Increased	Decreased	No data	Higher	Severe	Absent	[22]
*Paget’s disease*	Tibia	Higher	Lower	No data	Higher	Increased	Absent	[22]

**Table 3:** Overview of bony alterations of cortical structures in tuberculosis, syphilis, actinomycosis osteomyelitis and Paget’s disease in comparison to healthy bone (–: no information available)

Disease	Region	Thickness	Porosity	Trabecularization	Corticalis defect	Sclerosis	Spongy transformation	Reference
*Tuberculosis*	Vertebrae	Decreased	Increased	No difference	Increased	No difference	No difference	This study
*Tuberculosis*	Femur	Slight increase	Slight increase	Slight increase	Slight increase	Slight increase	Slight increase	This study
*Tuberculosis*	Tibia	Decreased	Increased	Increase	Slight increase	No difference	Increased	This study
*Syphilis*	Skull	Decreased	Increased	No data	Increased	No different	Not reported	[42]
*Actinomycosis*	Lumbar vertebrae	**-**	**-**	**-**	**-**	Reactive bone formation	**-**	[41]
*Actinomycosis*	Pelvic ring	**-**	**-**	**-**	**-**	Reactive bone formation	**-**	[41]
*Osteomyelitis*	Femur	Decreased	Increased	Increased	**-**	**-**	**-**	[23]
*Paget’s disease*	Femur	Increased	Increased	Increased	Absent	Absent	Absent	[22]
*Paget’s disease*	Tibia	Increased	Increased	Increased	Absent	Absent	Absent	[22]

As with tuberculosis, syphilis is a disease characterized by granuloma formation; Fraberger et al. assessed the microarchitecture of human skulls presenting signs of tertiary syphilis; although a different skeletal site than in this study was investigated, several alterations of the cortical compartment were similar (e.g. cortical thinning, cortical porosis, osteolysis [42]).

In Paget’s disease, similar as in tuberculosis, trabecular defects and cortical porosis were seen; nevertheless, in Paget’s disease cortical thickness was increased and no signs of ankylosis were detected [22], which is in contrast to tuberculosis.

In reviewing the abovementioned microstructural characteristics of several bone pathologies, it is obvious that there is no single pathognomonic feature of a specific disease; nevertheless, the combination of trabecular defects, decreased thickness, increased trabecular number and ankylosis could be suggestive of tuberculosis. Of note, alterations of the cortical compartment were less useful for a differential diagnosis of tuberculosis and other infectious diseases, whereas Paget’s disease showed relatively specific alterations of both compartments. Nevertheless, we think that in most cases a combination of findings and the consideration of the localization of the lesions will contribute to establishing a diagnosis.

Our study of historical tuberculosis specimens has some strengths as well as limitations. One limitation is that the diagnosis of tuberculosis is not substantiated by bacteriologic or molecular analyses but relies on typical macroscopic visible alterations. Nevertheless, the unpredictable state of DNA preservation in historic skeletal specimens very often constitutes technical challenges for ancient DNA diagnosis of *Mycobacterium tuberculosis* [43]. Moreover, it is possible that our samples represent relatively severe disease stages in a pre-antibiotic era, and may not reflect early, mild or treated cases. When comparing the finding in the femur and the tibiae, it needs to be taken into account that gender and age distributions were different.

On the other hand, the origin of our bones also implies several strengths. In general, the samples were obtained during pathology autopsies and thus allowed a synopsis of the autopsy findings and the observed bone alterations. Moreover, our samples are in an excellent state of preservation since they were prepared by maceration and then dry stored, preventing the modification of the bone structure by physical or chemical effects.

## Conclusion

This is one of the few studies that assessed the microstructure of skeletal sites affected by tuberculosis using microcomputed tomography. When compared to other bone pathologies, a combination of alterations at the trabecular compartment (trabecular defects, thinning of trabeculae, increased trabecular number and ankylosis) of historic skeletal remains, could substantiate the assumption that the individual had suffered from skeletal tuberculosis.

**Fig. 5:**
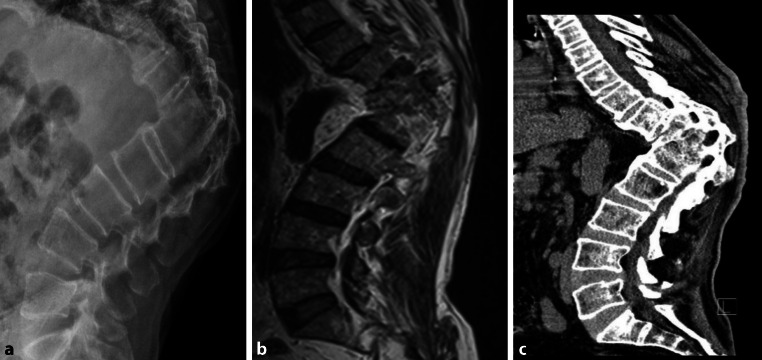
This 69-year-old male patient in 2020 was in inpatient care with a fracture of vertebral bodies due to tuberculosis infection. A 90° gibbus in the area of the thoracolumbar transition TH12–L1 with ankylosis and a Cobb angle of 45° is visible. **a** Conventional X‑ray. **b** Magnetic resonance imaging. **c** Conventional computed tomography

Because tuberculosis has still not been eradicated worldwide today, in everyday clinical practice one can still find patients in the last stage, skeletal tuberculosis stage, despite prevention and antibiotic treatment options (Fig. 5). If one sees spinal deformities in clinical practise one must not forget that the reason for this may be tuberculosis as an underlying disease. Thus, our study also underlines the relevance of collections such as the PASiN-NHM for contemporary medicine.

## Supplementary Information

Supplemental Tables 1–9
