# Supplementary material for: Microarchitecture of historic bone samples with tuberculosis
Source: Wien Klin Wochenschr. 2022 Mar 20;134(11-12):449–57. doi: 10.1007/s00508-022-02017-y (PMC8934580; doi:10.1007/s00508-022-02017-y)
Supplement: Supplementary file 1 — Supplemental Tables 1–9 [file 508_2022_2017_MOESM1_ESM.docx]

**Supplemental Material**

*Demography* Supplemental Tables 1-3

| **number** | **segment** | **year of death** | **sex** | **age at death** |
| --- | --- | --- | --- | --- |
| **1** | **L 1- 2** | **—** | **—** | **—** |
| **2** | **L 1-3** | **1822** | **female** | **25** |
| **3** | **L 2-5** | **—** | **—** | **—** |
| **4** | **L 4-5** | **—** | **—** | **—** |
| **5** | **Th12-L 3** | **—** | **—** | **—** |
| **6** | **L 3-5** | **—** | **—** | **—** |
| **7** | **L 4-5** | **1894** | **female** | **27** |
| **8** | **L 2** | **—** | **—** | **—** |
| **9** | **—** | **—** | **—** | **—** |
| **10** | **—** | **—** | **—** | **—** |
| **11** | **Th 10** | **—** | **—** | **—** |
| **12** | **Th 11-12** | **1926** | **male** | **37** |
| **13** | **L 1** | **1924** | **—** | **—** |
| **14** | **—** | **1822** | **female** | **25** |
| **15** | **L 1-5** | **—** | **—** | **—** |
| **16** | **L 5** | **—** | **—** | **—** |
| **17** | **L 5** | **1927** | **male** | **10** |
| **18** | **C 2** | **—** | **—** | **—** |
| **19** | **sacrum** | **—** | **—** | **—** |
| **20** | **sacrum** | **—** | **—** | **—** |

Supplemental Table 1: Biographical data of the individuals whose pathologically affected vertebrae were selected for sampling**,** C: cervical vertebra, Th: thoracic vertebra, L: lumbar vertebra, — : no data

| **number** | **side** | **year of death** | **sex** | **age at death** |
| --- | --- | --- | --- | --- |
| **1** | **left** | **—** | **—** | **—** |
| **2** | **right** | **—** | **—** | **—** |
| **3** | **right** | **1822** | **—** | **—** |
| **4** | **left** | **—** | **—** | **—** |
| **5** | **right** | **1821** | **male** | **23** |
| **6** | **right** | **1876** | **male** | **12** |
| **7** | **right** | **1840** | **male** | **28** |
| **8** | **left** | **1827** | **male** | **28** |
| **9** | **right** | **1827** | **—** | **—** |
| **10** | **left** | **—** | **—** | **—** |
| **11** | **right** | **—** | **—** | **—** |
| **12** | **right** | **1895** | **male** | **15** |
| **13** | **right** | **1937** | **male** | **66** |
| **14** | **left** | **1819** | **male** | **16** |
| **15** | **right** | **1819** | **male** | **19** |
| **16** | **right** | **—** | **male** | **25** |
| **17** | **left** | **—** | **—** | **—** |
| **18** | **left** | **1887** | **male** | **25** |
| **19** | **right** | **—** | **—** | **—** |

Supplemental Table 2: Biographical data of the individuals whose pathologically affected (right or left) femur was selected for sampling. **— :** no data

| **number** | **side** | **year of death** | **sex** | **age at death** |
| --- | --- | --- | --- | --- |
| **1** | **right** | **1937** | **female** | **74** |
| **2** | **—** | **—** | **—** | **—** |
| **3** | **left** | **—** | **—** | **—** |
| **4** | **left** | **—** | **—** | **—** |
| **5** | **left** | **—** | **—** | **—** |
| **6** | **left** | **—** | **—** | **—** |
| **7** | **—** | **—** | **—** | **—** |
| **8** | **—** | **1890** | **female** | **53** |
| **9** | **right** | **1920** | **—** | **—** |
| **10** | **right** | **1920** | **-** | **—** |
| **11** | **right** | **1901** | **female** | **67** |
| **12** | **left** | **—** | **—** | **—** |
| **13** | **—** | **1927** | **—** | **25** |
| **14** | **—** | **—** | **—** | **—** |
| **15** | **right** | **1889** | **male** | **32** |
| **16** | **—** | **—** | **—** | **—** |
| **17** | **left** | **—** | **—** | **—** |
| **18** | **left** | **—** | **—** | **—** |
| **19** | **left** | **—** | **—** | **—** |
| **20** | **left** | **—** | **—** | **—** |

Supplemental Table 3: Biographical data of the individuals whose pathologically affected (right or left) tibia was selected for sampling. **— :** no data

Supplemental Tables 4-9:

A representation of the semi-quantitative assessment of trabecular (Tb.) and of cortical (Ct.) bone structure in vertebral body, in femur and in tibia samples with tuberculosis.is shown in Supplemental Tables 4 – 9.

|  | **Tb.thickness** | **Tb.number** | **Sclerosis** | **Tb separation** | **Tb.defect** | **Ankylosis** |
| --- | --- | --- | --- | --- | --- | --- |
| **Vertebral b 1** | **- -** | **0** | **+** | **+** | **+** | **+** |
| **Vertebral b 2** | **- -** | **0** | **0** | **0** | **+** | **0** |
| **Vertebral b 3** | **- -** | **+** | **0** | **0** | **+ +** | **0** |
| **Vertebral b 4** | **- -** | **+ +** | **0** | **0** | **+ + +** | **0** |
| **Vertebral b 5** | **- -** | **+** | **0** | **+ +** | **+ +** | **+** |
| **Vertebral b 6** | **- - -** | **+ +** | **+** | **0** | **+ +** | **+** |
| **Vertebral b 7** | **-** | **0** | **0** | **0** | **+** | **+** |
| **Vertebral b 8** | **0** | **+** | **0** | **0** | **+ +** | **0** |
| **Vertebral b 9** | **0** | **+** | **+** | **0** | **+ +** | **+** |
| **Vertebral b 10** | **0** | **+** | **0** | **0** | **+ +** | **+** |
| **Vertebral b 11** | **- - -** | **+ + +** | **+ +** | **+** | **+ + +** | **+** |
| **Vertebral b 12** | **+** | **+** | **+** | **+** | **+ +** | **+ +** |
| **Vertebral b 13** | **0** | **0** | **+ +** | **0** | **+ +** | **+** |
| **Vertebral b 14** | **0** | **0** | **0** | **0** | **+ + +** | **0** |
| **Vertebral b 15** | **- -** | **++** | **+** | **+** | **+ +** | **0** |
| **Vertebral b 16** | **+** | **++** | **+ +** | **0** | **+ +** | **+ +** |
| **Vertebral b 17** | **- - -** | **-** | **+** | **0** | **+ +** | **0** |
| **Vertebral b 18** | **- -** | **-** | **+** | **0** | **+ +** | **0** |
| **Vertebral b 19** | **-** | **+ +** | **0** | **0** | **+ +** | **0** |
| **Vertebral b 20** | **- -** | **+** | **0** | **0** | **+ +** | **0** |
| **Median** | **- -** | **+** | **0/+** | **0** | **+ +** | **0/+** |

Supplemental Table.4: Semi-quantitative assessment of trabecular (Tb.) microarchitecture in vertebral body samples with tuberculosis.

| **Vertebral body samples** | **Ct.thickness** | **Ct.porosity** | **Trabecularisation** | **Ct.defect** | **Sclerosis** | **Spongy transformation** |
| --- | --- | --- | --- | --- | --- | --- |
| **1** | **- -** | **+ +** | **0** | **+** | **+** | **0** |
| **Vertebral b 2** | **-** | **+ +** | **0** | **+ +** | **0** | **0** |
| **Vertebral b 3** | **0** | **+ +** | **0** | **0** | **+** | **0** |
| **Vertebral b 4** | **- -** | **+ + +** | **0** | **+ +** | **0** | **0** |
| **Vertebral b 5** | **-** | **+ +** | **0** | **+ +** | **0** | **0** |
| **Vertebral b 6** | **- -** | **+ +** | **0** | **+ +** | **+** | **0** |
| **Vertebral b 7** | **- -** | **+ +** | **0** | **+ +** | **0** | **0** |
| **Vertebral b 8** | **-** | **+** | **0** | **+ +** | **0** | **0** |
| **Vertebral b 9** | **-** | **+** | **0** | **+ +** | **+ +** | **0** |
| **Vertebral b 10** | **- -** | **+ +** | **0** | **+ +** | **+** | **0** |
| **Vertebral b 11** | **- -** | **+ +** | **+ +** | **+** | **0** | **0** |
| **Vertebral b 12** | **0** | **+ +** | **+** | **+** | **+** | **0** |
| **Vertebral b 13** | **0** | **+ +** | **0** | **+** | **+** | **0** |
| **Vertebral b 14** | **- -** | **+ +** | **0** | **+ +** | **0** | **0** |
| **Vertebral b 15** | **-** | **+** | **0** | **+** | **+ +** | **0** |
| **Vertebral b 16** | **- -** | **+** | **0** | **+** | **+ +** | **0** |
| **Vertebral b 17** | **- -** | **+ +** | **0** | **+ +** | **0** | **0** |
| **Vertebral b 18** | **- -** | **+ +** | **0** | **+ +** | **0** | **0** |
| **Vertebral b 19** | **- -** | **+ +** | **0** | **+ +** | **0** | **0** |
| **Vertebral b 20** | **- -** | **+ + +** | **0** | **+** | **0** | **0** |
| **Median** | **- -** | **++** | **0** | **++** | **0** | **0** |

Supplemental Table.5: Semi-quantitative assessment of cortical (Ct.) microarchitecture in vertebral body samples with tuberculosis.

| **Semples** | **Tb. thickness** | **Tb.number** | **Sclerosis** | **Tb. separation** | **Tb.defect** | **Ankylosis** |
| --- | --- | --- | --- | --- | --- | --- |
| **Femur 1** | **- -** | **0** | **0** | **+** | **+** | **0** |
| **Femur 2** | **- - -** | **-** | **0** | **+** | **+ +** | **0** |
| **Femur 3** | **-** | **+** | **+** | **0** | **+** | **0** |
| **Femur 4** | **-** | **0** | **+** | **0** | **0** | **0** |
| **Femur 5** | **-** | **-** | **+** | **0** | **0** | **+ + +** |
| **Femur 6** | **0** | **+** | **0** | **0** | **+ +** | **+ + +** |
| **Femur 7** | **0** | **0** | **+** | **0** | **+** | **0** |
| **Femur 8** | **- -** | **+** | **+ +** | **+** | **+ +** | **+** |
| **Femur 9** | **- -** | **+ +** | **+** | **0** | **+** | **+ + +** |
| **Femur 10** | **0** | **0** | **+** | **0** | **0** | **0** |
| **Femur 11** | **- -** | **+** | **0** | **0** | **+** | **+ +** |
| **Femur 12** | **- - -** | **- -** | **0** | **+** | **+ +** | **0** |
| **Femur 13** | **+** | **+ +** | **+** | **0** | **+ +** | **+ + +** |
| **Femur 14** | **- -** | **- -** | **+** | **+** | **+ +** | **+ + +** |
| **Femur 15** | **+ +** | **- -** | **+ +** | **+** | **+ +** | **+ + +** |
| **Femur 16** | **+** | **+** | **+** | **+** | **+** | **+ + +** |
| **Femur 17** | **- - -** | **- -** | **+ +** | **-** | **+ +** | **+ + +** |
| **Femur 18** | **- - -** | **+ + +** | **+** | **+ +** | **+** | **+ + +** |
| **Femur 19** | **+ +** | **+** | **+ +** | **+** | **+ +** | **0** |
| **Median** | **-** | **0** | **+** | **+** | **+** | **+ +** |

Supplemental Table.6: Semi-quantitative assessment of trabecular (Tb.) microarchitecture in femur samples with tuberculosis.

| **Semples** | **Ct.thickness** | **Ct.porosity** | **Trabecularisation** | **Ct.defect** | **Sclerosis** | **Spongy transformation** |
| --- | --- | --- | --- | --- | --- | --- |
| **Femur 1** | **- -** | **+ +** | **0** | **+** | **0** | **+** |
| **Femur 2** | **- -** | **+ +** | **+** | **+ +** | **0** | **+** |
| **Femur 3** | **-** | **+** | **+** | **+** | **+** | **+** |
| **Femur 4** | **+ +** | **+ +** | **+ +** | **+ +** | **+** | **+** |
| **Femur 5** | **+ +** | **0** | **+** | **0** | **+** | **0** |
| **Femur 6** | **+ + +** | **+ +** | **+ +** | **+** | **0** | **+ +** |
| **Femur 7** | **+ + +** | **+** | **0** | **+** | **0** | **0** |
| **Femur 8** | **+** | **+ +** | **+** | **+** | **+ +** | **0** |
| **Femur 9** | **-** | **+** | **0** | **+** | **+** | **+** |
| **Femur 10** | **+** | **0** | **0** | **0** | **+** | **0** |
| **Femur 11** | **- - -** | **+ +** | **+ +** | **+** | **0** | **+** |
| **Femur 12** | **- -** | **+ +** | **+ +** | **+ +** | **0** | **+ +** |
| **Femur 13** | **+ +** | **0** | **0** | **0** | **+** | **0** |
| **Femur 14** | **-** | **+ +** | **0** | **+ +** | **0** | **+** |
| **Femur 15** | **+ +** | **+ +** | **0** | **+ +** | **0** | **+** |
| **Femur 16** | **+ +** | **0** | **0** | **+** | **+** | **+** |
| **Femur 17** | **+ +** | **+** | **+ +** | **+** | **0** | **+** |
| **Femur 18** | **-** | **+** | **+ + +** | **0** | **+** | **+ +** |
| **Femur 19** | **+ +** | **+** | **+ +** | **+** | **+** | **+** |
| **Median** | **+** | **+** | **+** | **+** | **+** | **+** |

.

Supplemental Table.7: Semi-quantitative assessment of cortical (Ct.) microarchitecture in femur samples with tuberculosis

| **Semples** | **Tb.thickness** | **Tb.number** | **Sclerosis** | **Tb. eparation** | **Tb.defect** | **Ankylosis** |
| --- | --- | --- | --- | --- | --- | --- |
| **Tibia 1** | **- - -** | **+ + +** | **+ +** | **+** | **+** | **0** |
| **Tibia 2** | **- - -** | **- -** | **0** | **0** | **+ +** | **+** |
| **Tibia 3** | **- -** | **+ + +** | **0** | **+** | **+ +** | **0** |
| **Tibia 4** | **- - -** | **+ +** | **+ +** | **0** | **+ +** | **0** |
| **Tibia 5** | **- - -** | **+ + +** | **0** | **0** | **+ +** | **0** |
| **Tibia 6** | **- -** | **+ +** | **+ +** | **0** | **+ +** | **+ +** |
| **Tibia 7** | **- - -** | **+ + +** | **+ +** | **0** | **+** | **+ + +** |
| **Tibia 8** | **- - -** | **+ + +** | **0** | **0** | **+ +** | **0** |
| **Tibia 9** | **- - -** | **+ + +** | **+ + +** | **0** | **0** | **0** |
| **Tibia 10** | **- - -** | **- -** | **0** | **+** | **+ +** | **0** |
| **Tibia 11** | **- - -** | **+ +** | **+** | **+** | **+ +** | **0** |
| **Tibia 12** | **- - -** | **+ + +** | **0** | **0** | **+ + +** | **0** |
| **Tibia13** | **- - -** | **+ + +** | **0** | **0** | **0** | **+ + +** |
| **Tibia14** | **- - -** | **+ + +** | **0** | **0** | **+ +** | **+ +** |
| **Tibia15** | **- - -** | **+ + +** | **+** | **0** | **+ +** | **0** |
| **Tibia 16** | **- - -** | **+ + +** | **0** | **0** | **0** | **0** |
| **Tibia17** | **- - -** | **+ + +** | **+ +** | **+ +** | **+ +** | **0** |
| **Tibia 18** | **- - -** | **+ +** | **0** | **0** | **+ + +** | **0** |
| **Tibia 19** | **-** | **+** | **+ +** | **0** | **+ +** | **0** |
| **Tibia 20** | **- - -** | **+ + +** | **+ +** | **+** | **+ +** | **0** |
| **Median** | **- - -** | **+ + +** | **0/+** | **0** | **+ +** | **0** |

Supplemental Table 8: Semi-quantitative assessment of trabecular (Tb.) microarchitecture in tibia samples with tuberculosis.

| **Semples** | **Ct.thickness** | **Ct.porosity** | **Trabecularisation** | **Ct. defect** | **Sclerosis** | **Spongy Transformation** |
| --- | --- | --- | --- | --- | --- | --- |
| **Tibia 1** | **+ + +** | **+** | **+** | **+** | **+** | **0** |
| **Tibia 2** | **- - -** | **+ +** | **+ +** | **0** | **0** | **0** |
| **Tibia 3** | **-** | **+ +** | **0** | **+ +** | **0** | **0** |
| **Tibia 4** | **- -** | **+ + +** | **+ +** | **+** | **+ +** | **+ +** |
| **Tibia 5** | **- -** | **+** | **+** | **0** | **0** | **0** |
| **Tibia 6** | **- - -** | **+** | **+ +** | **+ +** | **+** | **+** |
| **Tibia 7** | **- - -** | **+ + +** | **+ + +** | **+** | **+** | **+ +** |
| **Tibia 8** | **- - -** | **+** | **-** | **0** | **0** | **+ + +** |
| **Tibia 9** | **+ + +** | **+ + +** | **+ + +** | **0** | **+ + +** | **+ + +** |
| **Tibia 10** | **- -** | **+ +** | **+** | **+** | **+** | **+ +** |
| **Tibia 11** | **- -** | **+ +** | **+** | **+** | **+ +** | **+ +** |
| **Tibia 12** | **- - -** | **+ + +** | **+ + +** | **+ +** | **0** | **+ +** |
| **Tibia 13** | **- - -** | **+ + +** | **+ + +** | **+** | **0** | **0** |
| **Tibia 14** | **- - -** | **+ + +** | **+ +** | **0** | **0** | **+ +** |
| **Tibia 15** | **- - -** | **+ + +** | **+ +** | **+** | **0** | **+ +** |
| **Tibia 16** | **- - -** | **+ + +** | **+** | **0** | **0** | **+** |
| **Tibia 17** | **+ +** | **+ +** | **+ +** | **0** | **-** | **+ + +** |
| **Tibia 18** | **- - -** | **+ + +** | **+ + +** | **+ +** | **+ +** | **+ + +** |
| **Tibia 19** | **- -** | **+ +** | **+ + +** | **+ +** | **0** | **+** |
| **Tibia 20** | **- - -** | **+ + +** | **+ + +** | **+ + +** | **+ +** | **+ + +** |
| **Median** | **- - -** | **++ / +++** | **++** | **+** | **0** | **+ +** |

Supplemental Table 9: Semi-quantitative assessment of cortical (Ct.) microarchitecture in tibia samples with tuberculosis.
